# The Influence of Human Leukocyte Antigen and IL-10 Gene Polymorphisms on Hepatitis B Virus Outcome

**DOI:** 10.5812/hepatmon.6094

**Published:** 2012-05-30

**Authors:** Amitis Ramezani, Mohammad Banifazl, Setareh Mamishi, Masoomeh Sofian, Ali Eslamifar, Arezoo Aghakhani

**Affiliations:** 1 Clinical Research Department, Pasteur Institute of Iran, Tehran, IR Iran; 2 Pediatric Infectious Disease Research Center, Tehran University of Medical sciences, Tehran, IR Iran; 3 Iranian Society for Support of Patients with Infectious Disease, Tehran, IR Iran; 4 TPIRC (Tuberculosis and Pediatric Infectious Research Center), Arak University of Medical Sciences, Arak, IR Iran

**Keywords:** Hepatitis B Virus, Interleukin (IL)-10 gene, HLA Antigens

## Abstract

**Context:**

The clinical outcome of hepatitis B virus (HBV) infection is variable, ranging from spontaneous recovery to an inactive carrier state, chronic hepatitis, occult HBV infection, liver cirrhosis, or hepatocellular carcinoma.

**Evidence Acquisition:**

This variable pattern and clinical outcomes of the infection were mainly determined by virological and host genetic factors. Since the most of host genetic factors associated with HBV infection have currently focused on human leukocyte antigen (HLA) associations and interleukin (IL)-10 gene polymorphisms, this review focuses on the recent progresses in these issues to provide prognostic markers for the outcome of HBV infection.

**Results:**

A study on serum levels of IL-10 in occult HBV infected patients reported that the higher level of IL-10 production may suppress function of the immune system against HBV in patients with occult HBV infection. IL-10 promoter polymorphism at position -592 is associated with susceptibility to occult HBV infection.

**Conclusions:**

Findings of this study suggest that the host HLA polymorphism is an important factor in determining outcome of HBV infection but regarding IL-10 gene promoter polymorphisms, we are still have a long way to achieve a deﬁnite conclusion.

## 1. Context

Hepatitis B virus (HBV) infection is a major public health problem worldwide [1]. HBV is transmitted via contact with infected body fluids, including blood, saliva, and semen [2]. The outcome of HBV infection is highly variable, ranging from asymptomatic disease culminating in the spontaneous elimination of infection to persistent infection that can lead to occult HBV infection (OBI), cirrhosis, liver failure, or hepatocellular carcinoma [3][4]. The precise mechanisms leading to these various outcomes are not yet clearly defined, but environmental, immunological (such as the innate and adaptive immune responses against viral infection), virological (such as genotype and genetic divergence mutants), and host genetic factors are considered as important factors contribute in HBV infection outcome [3][5][6][7].

It has been perceived that host genetic factor is the critical component in determining the outcomes of HBV infection [1]. The most important host genetic factors associated with HBV infection outcome have currently focused on human leukocyte antigen (HLA) and cytokines associations [8]. Associations between HLA polymorphism and disease susceptibility as well as disease resistance have been well investigated, especially in autoimmune and metabolic diseases [9][10]. Besides extensive allele diversity in HLA is associated with susceptibility or protection of HBV infection in different ethnic populations [11].

Major histocompatibility complex (MHC) gene products are vital in regulating several antiviral immune reactions. In addition, genetic factors controlling host immune response could play an important role in determining infection outcome. Alloantigens are taken up by antigen-presenting cells, which process them and re-express the antigens on the cell surface along with HLAs to be recognized by the T-cell receptor. The polymorphisms of HLA alleles may cause significant changes in the presentation of antigen to T cell receptor, which in turn affects immune response [12][13]. Several proinflammatory cytokines such as T helper (Th) 1 cytokines (including interleukin (IL)-2 and interferon (IFN)-gamma) have been identified to participate in the process of viral clearance and host immune response to HBV. In contrast, the Th2 cytokine (IL-10) serves as a potent inhibitor of Th1 effector cells in HBV diseases. Polymorphisms in the regulatory regions of the cytokine genes may influence their expression. Therefore, as genetic predictors of disease susceptibility or clinical outcome, the polymorphisms of cytokine genes are potentially important [8][14].

In the last decade, the virological and immunological factors associated with HBV infection outcome have been extensively studied, but the examination of the relationship between host genetics and HBV resistance is still scarce [8][15][16]. Since the most of host genetic factors associated with HBV infection have currently focused on HLA associations and IL-10 gene polymorphisms, this review focuses on the recent progresses in these issues.

## 2. Evidence Acquisition

### 2.1. HLA Class I and II Alleles

Highly polymorphic HLA gene (a cluster of closely linked genes including class I, class II, and class III) is located on the short arm of chromosome 6 [17] and play a critical role in modulation of immune response. Effective presentation of viral antigens to CD4+ T cells and CD8+ T cells by HLA class II and I molecules respectively, is the key regulation of optimum immune response against viral infection and further dictates of viral clearance or persistence [11][18]. Due to the specific antigen presenting function of HLA in immune responsiveness, the contribution of HLA to the outcome of HBV infection has been studied in different populations. However, data have shown some inconsistencies with regard to HLA effects on HBV clearance or persistence in different ethnic and racial groups [7].

Both HLA class I and II alleles have been associated with the outcome of HBV infection, although the majority of studies have focused on the associations between HLA class II alleles and HBV infection [19][20]. Chen et al. [21] showed that allele frequencies of HLA-B8, DR3, A30, and DQA1*0501 were high in patients with chronic hepatitis B (CHB) infection, suggesting that these alleles are associated with CHB. A study in African-American adults reported that HBV persistence was correlated with DQA1*0501 and DQB1*0301 alleles found in a common DQA1-DQB1 haplotype [22]. Thio et al. [18] reported that HLA-B*08 was associated with viral persistence.

The data of Yang indicated that HLA-DRB1*03 and HLADRB1*07 were related to susceptibility to chronic HBV infection, and that DRB1*15 was negatively related to the persistence of chronic HBV infection among people in northwestern China [1], whereas Meng et al. [23] showed that HLA-DRB1*1201 is associated with protection against chronic hepatitis B, and HLA-DR9 and DQ9 are associated with chronicity of HBV infection in Zhejiang Province, China. A study in northern Chinese patients showed that susceptibility to chronic hepatitis B was strongly associated with HLA-DRB1*10 allele [24]. The findings of Jiang suggested that HLA-DRB1*0301, DQA1*0501, and DQB1*0301 are closely associated with the susceptibility to CHB, and HLA-DRB1*1101/1104 and DQA1*0301 are related to resistance to CHB [26]. Han et al. [25] reported that HLA-DRB1*06, -DRB1*08, and -DRB1*16 may be associated with chronicity of HBV infection, and HLA-DRB1*07 with protection against HBV infection.

Hwang et al. [26] reported the associations between HLA-A33 or DR7 and HBV chronicity. Their study strongly showed that a combination of these two antigens has an additive effect on HBV persistency. Another Korean study found that the presence of DRB1*0701 and DQB1*0301 was associated with chronic HBV infection [7]. In a study involving subjects from eastern Turkey, frequencies of HLA-B35, -CW4, -DQ2, and -DQ8 were markedly higher in chronic HBV group than those in spontaneously recovered group [3]. A study by Ramezani et al. [19] in Iran showed that the frequency of HLA-A*33 allele was higher in HBV persistent group whereas the frequency of the DRB1*13 allele was higher in HBV recovered group. They reported that HLA-A*3303 and DRB1*1301 were predominant allelic subtypes related to HBV infection outcomes [27].

Thursz et al. [20] studied both children and adults in Gambia, and found that MHC class II allele HLA-DRB1*1302 was more frequent in individuals that had cleared the infection than in those presenting persistent infection. Cotrina and Thio et al. [18][28] also showed that HLA-DRB1*1301 and -DRB1*1302 alleles were associated with the clearance of HBV infection and protected people against chronic hepatitis B. Karan and Diepolder et al. [29][30] also observed that HLA-DR13 was present at a higher ratio in the group capable of creating spontaneous HBV antibody, suggesting that HLA-DR13 is associated with a self-course of HBV infection. Ahn et al. [31] reported that HLA-DR6 and especially HLA-DR13 are associated with the elimination of the HBV infection. In a Taiwanese study, HLA-DRB1*0406 was associated with recovery from HBV infection in Han Chinese, as was HLA-B*4001 in Aborigines [32]. Studies investigating HLA association with hepatitis B infection outcome are summarized in Table 1.

**Table 1:** Studies Investigating HLA Association With Hepatitis B Infection Outcome

	**Country**	**Persistent HBV Infection**	**Self Limited HBV Infection**
Thursz *et al.* [20] (1995)	Gambia	-	HLA-DRB1*1302
Chen *et al.* [21] ( 1996)	Caucasians	HLA-B8, A30, DR3, DQA1*0501	-
Thio *et al.* [18] (2003)	Caucasians	HLA-B*08	HLA-A*0301,DRB1*1302
Cotrina *et al*.[28] (1997)	Spain	-	HLA-DRB1*1301 , DRB1*1302
Hohler *et al*. [44] (2005)	Germany	-	HLA-DRB1* 1301, DRB1* 1302
Diepolder *et al. *[29] (1998)	Germany	-	HLA-DR13
Thio *et al*.[22] (1999)	African American	HLA-DQA1*0501, DQB1*0301	-
Shen *et al*.[24] (1999)	China	HLA-DRB1*10	-
Jiang *et al*.[59] (2003)	China	HLA-DRB1*0301, DQA1*0501, DQB1*0301	HLA-DRB1*1101/1104, DQA1*0301
Meng *et al. *[23] (2003)	China	HLA-DR9 , DQ9	HLA-DRB1*1201
Han *et al.* [25] (2005)	China	HLA-DRB1*06, DRB1*08, DRB1*16	HLA-DRB1*07
Zhang *et al. *[54] (2006)	China	HLA-DRB1*12	HLA-A*02
Xi-Lin *et al. *[60] (2006)	China	-	HLA-DQB1*0503, *0303
Lu *et al.*[61] (2006)	China	HLA-DQA1 * 0302	HLA-DQA1 * 0102HLA-DQA1 * 0301
Yang *et al*.[1] (2007)	China	HLA-DRB1*03, DRB1*07	HLA- DRB1*15
Zhu *et al.* [17][62] (2007)	China	HLA-DQB1*0502	-
Song *et al.* [62] (2007)	China	HLA-DRB1*0401	-
Liu *et al.* [63](2007)	China	HLA-DQB1*0201, DQA1*0601, DQB1*0601, DQA1*0201	HLA-DQA1*0102 , DQA1*0104
Singh *et al.*[11] (2007)	China	HLA-DQA1*0501, HLA-DQB1*0301	
Li *et al.* [64](2011)	China	HLA-DRB1*07, 12	HLA-B*51,B*15, DRB1*11 , 14
Ahn *et al.*[31] (2000)	Korea	-	HLA-DR6, DR13
Hwang *et al.*[26] (2007)	Korea	HLA-A33, DR7	-
Cho *et al.* [7] (2008)	Korea	HLA-DRB1*0701, DQB1*0301	HLA-DRB1*1302, DQB1* 0609
Karan *et al*.[30] (2002)	Turkey	-	HLA-DR13
Albayrak *et al*.[3] (2011)	Turkey	HLA-B35, CW4, DQ2,DQ8	-
Kummee *et al.*[65](2007)	Thailand	HLA-DR12	HLA-DR13
Ramezani *et al*.[27] (2009)	Iran	HLA-A*3303	HLA-DRB1*1301
Fletcher *et al*. [66] (2011)	India	HLA-DRB1*0701, B*44	HLA-DRB1*0301

### 2.2. IL-10 Gene Polymorphisms

Cytokines, mainly secreted by lymphocytes and monocytes, serve as immune response molecules with various physiological functions that regulate immunologic, inflammatory, and reparative host responses . T cell derived cytokines are important in the host immune response [33]. Activated T lymphocytes are divided into two functional subsets, Th l and Th2 cells, on the basis of cytokines that they produce [33]. Thl cytokines, including IL-2, IFN-gamma, and tumor necrosis factor (TNF)-α, are involved principally in cell-mediated immunity and play a crucial role in protection against intracellular pathogen [33][34], whereas Th2 cytokines, including IL-4, IL-5, and IL-10, mostly regulate antibody mediated immunity; their effects can be beneficial against extracellular agents but can be associated with progressive disease by intracellular pathogens [34][35]. Interleukin-10 is one of the critical modulators which is produced by activated macrophages, T regulatory, B regulatory, and Th2 lymphocytes and inhibits expression of other proinflammatory cytokines such as IFN-gamma, IL-2, and TNF-alpha in Th1 cells [36]. IL-10 influences natural history of HBV infection and other viral diseases such as Epstein-Barr, herpes zoster, HIV, and hepatitis C [37][38][39]. The levels of IL-10 production determine immune regulation and the balance between inflammatory and anti-inflammatory responses [4].

There are some evidences showing that the capacity of IL-10 production is a major genetic component which has an association with development and progression of HBV infection [40][41][42]. This has been ascribed to polymorphisms within the regulatory regions or signal sequences of this cytokine gene which have shown to be important in the susceptibility to inflammatory disease [8][41], responsiveness to HBV vaccination [43], HBeAg seroconversion in HBV carriers [44] and HBV-related hepatocellular carcinoma [45][46]. In IL-10 gene promoter region, three biallelic polymorphisms were determined at positions -1082, -819, and -592 [8]. The carriers of -1082 G/G genotype are high IL-10 producers, the -1082 G/A carriers show intermediate production of IL-10, and the -1082 A/A genotype is associated with low IL-10 production. Polymorphisms at position -819 and -592 have no independent influence on IL-10 production [47].

IL-10-819 T and C alleles were completely in linkage disequilibrium with IL-10-592A and C alleles, respectively. The -592A allele was exclusively associated with the-1082A allele. These result in three different haplotypes: GCC, ACC and ATA [48]. Miyazoe et al. [41] analyzed the distributions of IL-10 promoter single nucleotide polymorphisms (SNPs) in Japanese HBV-infected patients and found that the -819T and -592A wild-type alleles in IL-10 gene promoter were significantly more common in asymptomatic carriers than in patients with chronic progressive liver diseases, suggesting that inheritance of IL-10 gene promoter polymorphisms is relevant to progression in chronic HBV infection, perhaps due to decreased IL-10 production induced by -819T and -592A alleles. Turner et al. [48] also indicated that A/A genotype at position -592 in IL-10 gene promoter, which was associated with lower IL-10 levels, had a modest effect in HBV clearance. Shin et al. [46] reported that in high IL-10 producers, chronic HBV infection progression accelerated. In contrast Cheong et al. [49] reported that C/C genotype frequencies at position −592 in IL-10 gene promoter were significantly higher in HBV clearance group than in HBV persistence group. They suggested that the carriers of IL-10-592 C/C (high IL-10 producers) were more likely to clear HBV spontaneously compared to those carrying IL-10-592A allele (low IL-10 producers).

A meta analysis conducted by Lu et al. [50] indicated that the G and A alleles in IL-10-1082 were not associated with HBV infection in Asian population, whereas another meta analysis in Chinese pooled population reported a relationship between IL-10-1082G/A polymorphisms and persistent HBV infection and indicated that there was significantly reduced risk of persistent HBV infection with IL-10-1082A/A genotypes.

## 3. Results

The results of this study suggest that carriers of IL-10-592A allele were more likely to clear HBV spontaneously [2]. Another study in African Americans also showed that HBV infection outcome was influenced by proximal promoter SNP IL10-1082 [51]. Gao et al. [4] showed that IL-10-1082 A/A was associated with an increased risk, but-1082 A/G with a reduced risk of persistent HBV infection. In an investigation by Peng et al. [45] patients with intermediate producer genotypes or haplotypes of IL-10 had more ability to produce anti-HBe than those with low producer genotypes or haplotypes, so they were usually associated with a covert mode of HBeAg seroconversion. Another study by Wu et al. [52] showed that patients with G/G genotype in the −1082 promoter area of IL-10 gene underwent HBeAg seroconversion at a significantly younger age than patients who had G/A and A/A genotypes. They suggested that a polymorphism at the −1082 locus of IL-10 gene promoter may modulate production of IL-10, and G/G genotype is considered as a high-production genotype compared to G/A and A/A genotypes.

Yan et al. [53] demonstrated that the -592C allele and the -1082A-819C-592C haplotype in IL-10 gene promoter were associated with an increased susceptibility to acute liver failure in HBV carriers. The study by Zhang et al. [54] showed no significant difference in frequencies of genotypes and alleles of IL-10 gene promoter region at position -1082 G/A, -819 T/C, -592 A/C among normal controls, individuals spontaneously recovering from HBV infection, and patients with chronic hepatitis B. However, they reported that frequencies of T/T genotype at position -819 and A/A genotype at position -592 in chronic hepatitis B were significantly higher than that in asymptomatic HBV carriers. Li et al. [55] also showed no significant differences in -1082, -819 and -592 of IL-10 gene between cases and healthy controls. An investigation in Iran is also found no significant difference in frequencies of genotypes and haplotypes of IL-10 gene promoter region at position -1082, -819 and -592 among cases with HBV infection and controls but they reported that frequencies of A/A genotype at position -592 and T/T genotype at position -819 were higher in HBV clearance group, while frequency of G/G genotype at position -1082 was higher in persistence group. GCC/GCC and GCC/ACC haplotypes were significantly more frequent in anti-HBe positive patients [56].

## 4. Conclusions

Hepatitis B virus is the most common cause of acute and chronic liver disease worldwide. Chronic HBV infection is a multifactorial disease which is related to viral genotypes and host genetic factors. Recent studies have shown that HLA and cytokine genetic polymorphisms have an association with the development of chronic HBV infection and the progression of the infection. We have aimed to review the published literature on polymorphisms of HLA and IL-10 gene promoter associations with HBV infection outcome with the purpose to provide prognostic markers for the outcome of HBV infection.

## References

[1] Yang G, Liu J, Han S, Xie H, Du R, Yan Y (2007). Association between hepatitis B virus infection and HLA‐DRB1 genotyping in Shaanxi Han patients in northwestern China. Tissue Antigens.

[2] Zhang TC, Pan FM, Zhang LZ, Gao YF, Zhang ZH, Gao J (2011). A meta-analysis of the relation of polymorphism at sites -1082 and-592 of the IL-10 gene promoter with susceptibility and clearance to persistent hepatitis B virus infection in the Chinese population. Infection.

[3] Albayrak A, Ertek M, Tasyaran MA, Pirim I (2011). Role of HLA allele polymorphism in chronic hepatitis B virus infection and HBV vaccine sensitivity in patients from eastern Turkey. Biochem Genet.

[4] Gao QJ, Liu DW, Zhang SY, Jia M, Wang LM, Wu LH (2009). Polymorphisms of some cytokines and chronic hepatitis B and C virus infection. World J Gastroenterol.

[5] Aghakhani A, Hamkar R, Zamani N, Eslamifar A, Banifazl M, Saadat A (2009). Hepatitis B virus genotype in Iranian patients with hepatocellular carcinoma. Int J Infect Dis.

[6] Chisari FV, Ferrari C (1995). Hepatitis B virus immunopathogenesis. Annu Rev Immunol.

[7] Cho SW, Cheong JY, Ju YS, Oh do H, Suh YJ, Lee KW (2008). Human leukocyte antigen class II association with spontaneous recovery from hepatitis B virus infection in Koreans: analysis at the haplotype level. J Korean Med Sci.

[8] Wang FS (2003). Current status and prospects of studies on human genetic alleles associated with hepatitis B virus infection. World J Gastroenterol.

[9] Donaldson PT, Doherty DG, Hayllar KM, McFarlane IG, Johnson PJ, Williams R (1991). Susceptibility to autoimmune chronic active hepatitis: human leukocyte antigens DR4 and A1-B8-DR3 are independent risk factors. Hepatology.

[10] Gibbons GH, Pratt RE, Dzau VJ (1992). Vascular smooth muscle cell hypertrophy vs. hyperplasia. Autocrine transforming growth factor-beta 1 expression determines growth response to angiotensin II. J Clin Invest.

[11] Singh R, Kaul R, Kaul A, Khan K (2007). A comparative review of HLA associations with hepatitis B and C viral infections across global populations. World J Gastroenterol.

[12] Peano G, Menardi G, Ponzetto A, Fenoglio LM (1994). HLA-DR5 antigen. A genetic factor influencing the outcome of hepatitis C virus infection?. Arch Intern Med.

[13] Racioppi L, Ronchese F, Schwartz RH, Germain RN (1991). The molecular basis of class II MHC allelic control of T cell responses. J Immunol.

[14] Fiorentino DF, Zlotnik A, Vieira P, Mosmann TR, Howard M, Moore KW (1991). IL-10 acts on the antigen-presenting cell to inhibit cytokine production by Th1 cells. J Immunol.

[15] Dean M, Carrington M, O’Brien SJ (2002). Balanced polymorphism selected by genetic versus infectious human disease. Annu Rev Genomics Hum Genet.

[16] Hill AV (1998). Host genetics of infectious diseases: old and new approaches converge. Emerg Infect Dis.

[17] Zhu XL, Du T, Li JH, Lu LP, Guo XH, Gao JR (2007). Association of HLADQB1 gene polymorphisms with outcomes of HBV infection in Chinese Han population. Swiss Med Wkly.

[18] Thio CL, Thomas DL, Karacki P, Gao X, Marti D, Kaslow RA (2003). Comprehensive analysis of class I and class II HLA antigens and chronic hepatitis B virus infection. J Virol.

[19] Ramezani A, Hasanjani Roshan MR, Kalantar E, Eslamifar A, Banifazl M, Taeb J (2008). Association of human leukocyte antigen polymorphism with outcomes of hepatitis B virus infection. J Gastroenterol Hepatol.

[20] Thursz MR, Kwiatkowski D, Allsopp CE, Greenwood BM, Thomas HC, Hill AV (1995). Association between an MHC class II allele and clearance of hepatitis B virus in the Gambia. N Engl J Med.

[21] Chen DF, Kliem V, Endres W, Brunkhorst R, Tillmann HL, Koch KM (1996). Relationship between human leukocyte antigen determinants and courses of hepatitis B virus infection in Caucasian patients with end-stage renal disease. Scand J Gastroenterol.

[22] Thio CL, Carrington M, Marti D, O’Brien SJ, Vlahov D, Nelson KE (1999). Class II HLA alleles and hepatitis B virus persistence in African Americans. J Infect Dis.

[23] Meng XQ, Chen HG, Ma YL, Liu KZ (2003). Influence of HLA class II molecules on the outcome of hepatitis B virus infection in population of Zhejiang Province in China. Hepatobiliary Pancreat Dis Int.

[24] Shen J, Ji Y, Guan X, Huang R, Sun Y (1999). The association of HLA-DRBI* 10 with chronic hepatitis B in Chinese patients. Chinese J Microb Immun.

[25] Han YN, Yang JL, Zheng SG, Tang Q, Zhu W (2005). Relationship of human leukocyte antigen class II genes with the susceptibility to hepatitis B virus infection and the response to interferon in HBVinfected patients. World J Gastroenterol.

[26] Hwang SH, Sohn YH, Oh HB, Hwang CY, Lee SH, Shin ES (2007). Human leukocyte antigen alleles and haplotypes associated with chronicity of hepatitis B virus infection in Koreans. Arch Pathol Lab Med.

[27] Ramezani A, Aghakhani A, Kalantar E, Banifazl M, Eslamifar A, Velayati AA (2009). HLA-A *3303* and *3301 predispose patients to persistent hepatitis B infection. J Gastrointestin Liver Dis.

[28] Cotrina M, Buti M, Jardi R, Rodriguez-Frias F, Campins M, Esteban R (1997). [Study of HLA-II antigens in chronic hepatitis C and B and in acute hepatitis B].. Gastroenterol Hepatol.

[29] Diepolder HM, Jung MC, Keller E, Schraut W, Gerlach JT, Gruner N (1998). A vigorous virus-specific CD4+ T cell response may contribute to the association of HLA-DR13 with viral clearance in hepatitis B. Clin Exp Immunol.

[30] Karan MA, Tascioglu NE, Ozturk AO, Palanduz S, Carin M (2002). The role of HLA antigens in chronic hepatitis B virus infection. J Pak Med Assoc.

[31] Ahn SH, Han KH, Park JY, Lee CK, Kang SW, Chon CY (2000). Association between hepatitis B virus infection and HLA-DR type in Korea. Hepatology.

[32] Wu YF, Wang LY, Lee TD, Lin HH, Hu CT, Cheng ML (2004). HLA phenotypes and outcomes of hepatitis B virus infection in Taiwan. J Med Virol.

[33] Rico MA, Quiroga JA, Subira D, Castanon S, Esteban JM, Pardo M (2001). Hepatitis B virus-specific T-cell proliferation and cytokine secretion in chronic hepatitis B e antibody-positive patients treated with ribavirin and interferon alpha. Hepatology.

[34] Guidotti LG, Ando K, Hobbs MV, Ishikawa T, Runkel L, Schreiber RD (1994). Cytotoxic T lymphocytes inhibit hepatitis B virus gene expression by a noncytolytic mechanism in transgenic mice. Proc Natl Acad Sci U S A.

[35] Fishman MA, Perelson AS (1994). Th1/Th2 cross regulation. J Theor Biol.

[36] Pestka S, Krause CD, Sarkar D, Walter MR, Shi Y, Fisher PB (2004). Interleukin-10 and related cytokines and receptors. Annu Rev Immunol.

[37] Oleksyk TK, Thio CL, Truelove AL, Goedert JJ, Donfield SM, Kirk GD (2005). Single nucleotide polymorphisms and haplotypes in the IL10 region associated with HCV clearance. Genes Immun.

[38] Shin HD, Winkler C, Stephens JC, Bream J, Young H, Goedert JJ (2000). Genetic restriction of HIV-1 pathogenesis to AIDS by promoter alleles of IL10. Proc Natl Acad Sci U S A.

[39] Vicari AP, Trinchieri G (2004). Interleukin-10 in viral diseases and cancer: exiting the labyrinth?. Immunol Rev.

[40] Ben-Ari Z, Mor E, Papo O, Kfir B, Sulkes J, Tambur AR (2003). Cytokine gene polymorphisms in patients infected with hepatitis B virus. Am J Gastroenterol.

[41] Miyazoe S, Hamasaki K, Nakata K, Kajiya Y, Kitajima K, Nakao K (2002). Influence of interleukin-10 gene promoter polymorphisms on disease progression in patients chronically infected with hepatitis B virus. Am J Gastroenterol.

[42] Mosmann TR (1994). Properties and functions of interleukin-10. Adv Immunol.

[43] Bidwell J, Keen L, Gallagher G, Kimberly R, Huizinga T, McDermott MF (1999). Cytokine gene polymorphism in human disease: on-line databases. Genes Immun.

[44] Hohler T, Reuss E, Freitag CM, Schneider PM (2005). A functional polymorphism in the IL-10 promoter influences the response after vaccination with HBsAg and hepatitis A. Hepatology.

[45] Peng XM, Huang YS, Ma HH, Gu L, Xie QF, Gao ZL (2006). Interleukin-10 promoter polymorphisms are associated with the mode and sequel of HBeAg seroconversion in patients with chronic hepatitis B virus infection. Liver Int.

[46] Shin HD, Park BL, Kim LH, Jung JH, Kim JY, Yoon JH (2003). Interleukin 10 haplotype associated with increased risk of hepatocellular carcinoma. Hum Mol Genet.

[47] Tseng LH, Lin MT, Shau WY, Lin WC, Chang FY, Chien KL (2006). Correlation of interleukin-10 gene haplotype with hepatocellular carcinoma in Taiwan. Tissue Antigens.

[48] Turner DM, Williams DM, Sankaran D, Lazarus M, Sinnott PJ, Hutchinson IV (1997). An investigation of polymorphism in the interleukin-10 gene promoter. Eur J Immunogenet.

[49] Cheong JY, Cho SW, Hwang IL, Yoon SK, Lee JH, Park CS (2006). Association between chronic hepatitis B virus infection and interleukin-10, tumor necrosis factor-alpha gene promoter polymorphisms. J Gastroenterol Hepatol.

[50] Lu YL, Wu X, Huang HL, Dai LC (2010). Allele polymorphisms of interleukin-10 and hepatitis B, C virus infection. Chin Med J (Engl)..

[51] Truelove AL, Oleksyk TK, Shrestha S, Thio CL, Goedert JJ, Donfield SM (2008). Evaluation of IL10, IL19 and IL20 gene polymorphisms and chronic hepatitis B infection outcome. Int J Immunogenet.

[52] Wu JF, Ni YH, Lin YT, Lee TJ, Hsu SH, Chen HL (2011). Human interleukin-10 genotypes are associated with different precore/core gene mutation patterns in children with chronic hepatitis B virus infection. J Pediatr.

[53] Yan Z, Tan W, Zhao W, Dan Y, Wang X, Mao Q (2009). Regulatory polymorphisms in the IL-10 gene promoter and HBV-related acute liver failure in the Chinese population. J Viral Hepat.

[54] Zhang PA, Li Y, Yang XS (2006). [Associated study on interleukin 10 gene promoter polymorphisms related to hepatitis B virus infection in Chinese Han population].. Zhonghua Yi Xue Yi Chuan Xue Za Zhi.

[55] Li C, Zhi-Xin C, Li-Juan Z, Chen P, Xiao-Zhong W (2006). The association between cytokine gene polymorphisms and the outcomes of chronic HBV infection. Hepatol Res.

[56] Kalantar E, Ramezani A, Hosseini S, Aghakhani A, Zarin Far N, Banifazl M (2011). [Evaluation of Interleukin-10 Gene Promoter Polymorphisms and Hepatitis B Virus Infection Outcome].. Iran J Infec Dis Trop Med.

[57] Arababadi MK, Pourfathollah AA, Jafarzadeh A, Hassanshahi G (2010). Serum Levels of IL-10 and IL-17A in Occult HBV-Infected South-East Iranian Patients. Hepat Mon.

[58] Ahmadabadi BN, Hassanshahi G, Arababadi MK, Leanza C, Kennedy D (2012). The IL-10 Promoter Polymorphism at Position -592 is Correlated with Susceptibility to Occult HBV Infection. Inflammation.

[59] Jiang YG, Wang YM, Liu TH, Liu JB (2003). Association between HLA class II gene and susceptibility or resistance to chronic hepatitis. World J Gastroenterol.

[60] Xi-Lin Z, Te D, Jun-Hong L, Liang-Ping L, Xin-Hui G, Ji-Rong G (2006). Analysis of HLA-DQB1 gene polymorphisms in asymptomatic HBV carriers and chronic hepatitis B patients in the Chinese Han population. Int J Immunogenet.

[61] Lu LP, Liu Y, Li XW, Sun GC, Zhu XL, Wu YZ (2006). [Association of polymorphisms of human leucocyte antigen -DRB1 and -DQA1 allele with outcomes of hepatitis B virus infection in Han population of north China].. Zhongguo Yi Xue Ke Xue Yuan Xue Bao.

[62] Song MS, Li HW, Peng HY, Duan BN, Chen H, Xu LQ (2007). [Association of polymorphism on HLA-DRB1*04 alleles with outcome of hepatitis B virus infection].. Zhonghua Yi Xue Yi Chuan Xue Za Zhi.

[63] Liu C, Cheng B (2007). Association of polymorphisms of human leucocyte antigen-DQA1 and DQB1 alleles with chronic hepatitis B virus infection, liver cirrhosis and hepatocellular carcinoma in Chinese. Int J Immunogenet.

[64] Li J, Yang D, He Y, Wang M, Wen Z, Liu L (2011). Associations of HLADP variants with hepatitis B virus infection in southern and northern Han Chinese populations: a multicenter case-control study. PLoS One.

[65] Kummee P, Tangkijvanich P, Poovorawan Y, Hirankarn N (2007). Association of HLA-DRB1*13 and TNF-alpha gene polymorphisms with clearance of chronic hepatitis B infection and risk of hepatocellular carcinoma in Thai population. J Viral Hepat.

[66] Fletcher GJ, Samuel P, Christdas J, Gnanamony M, Ismail AM, Anantharam R (2011). Association of HLA and TNF polymorphisms with the outcome of HBV infection in the South Indian population. Genes Immun.

